# Single atom catalysts push the boundaries of heterogeneous catalysis

**DOI:** 10.1038/s41467-021-26130-0

**Published:** 2021-10-11

## Abstract

Single atom catalysts hold the potential to significantly impact the chemical and energy industrial sectors. This editorial introduces the state of the field along with a collection of Articles and Comments that encapsulate the ongoing efforts of the research community in this field.

The catalysis community has long utilized inorganic or organometallic complexes with well-defined transition metal centers in tailored coordination environments to drive catalytic reactions in homogeneous solutions. Contemporary research in heterogeneous catalysis has broadly taken inspiration from these approaches and has focused intensely on maximizing the concentration of active catalytic sites to improve reaction efficacy and minimize metallic waste. To this end, the identification of single-atom catalysts (SACs) of transition metal elements embedded on or within heterogeneous substrates has been transformative. Single-atom sites, however isolated, can exist in many different coordination environments, all of which have important roles in defining catalytic activity and selectivity toward a product. Indeed, since the early work of Flytzani-Stephanopoulos and others^1^ on this motif, the field has grown quickly (Fig. 1). This is due, in part, to the advent of sophisticated imaging and x-ray spectroscopic techniques. Such technologies are helping researchers capture vivid images of the SACs on a substrate surface even during a chemical reaction. Meanwhile, modern synthetic methodologies are now able to achieve high yields of carefully designed SAC environments with targeted applications across chemical catalysis.

**Fig. 1:**
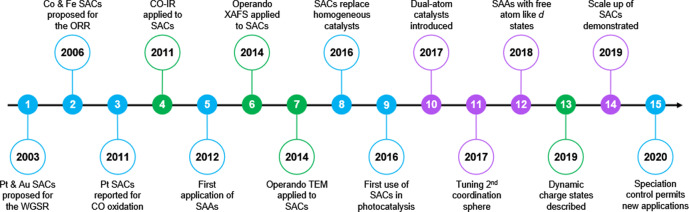
The journey of single-atom catalysts^2^.

It is now clear that single-atom catalytic sites have rightfully earned the attention of many scientists in the catalysis community and beyond because of promising intrinsic characteristics such as high metal dispersion and activity, plus targeted product selectivity that helps to bridge the gap between homogeneous and heterogeneous catalysis. Equally exciting are emerging examples of SAC-based materials that are now beginning to span across a broad spectrum of catalytic applicability and detailed mechanistic understanding.

To capture some exciting results in this vibrant field, here we showcase a collection of studies published in *Nature Communications* that highlight recent advances in the diverse field of SACs. The selected works range from general and scalable synthetic methodologies to laboratory-scale reactions with fundamental mechanistic and theoretical insights. Also featured are some articles that report on SAC candidates for industrially relevant catalytic reactions with long-term stability and exceptional selectivity toward target products at large scales.

To capture some exciting results in this vibrant field, here we showcase a collection of studies published in *Nature Communications* that highlight recent advances in the diverse field of single-atom catalysts.

Stunning progress has been made in the field of SACs within the past decade and there is a clear promise of a bright future for these materials. Mitchell and Pérez-Ramírez^2^ overview the journey of SACs in their recent comment and identify several emerging frontiers for future research (Fig. 1). In another comment, Datye and Guo^3^ further discuss the transition of SACs research from academic curiosity to industrial deployment.

Nevertheless, large-scale and universal synthesis of SACs is likely necessary before achieving feasible industrial applications. Over the years, many interesting synthetic approaches have been developed for the general and large-scale synthesis of SACs. Among them, the precursor-dilution strategy^4^ exhibits remarkable adjustability and generality, providing sufficient freedom to design SACs catalysts on the atomic scale. Another methodology is ligand-mediated^5^, which features both high universality and scalability (>1 kg scale). In addition to the generality and scalability, the precise engineering of the local coordination of the single atom to optimize catalytic activity is similarly important. In this regard, Shang et al.^6^ has explored a three-step process to construct Cu-S1N3 single-atom sites with enhanced oxygen reduction activity.

Improving the fundamental understanding of SACs will facilitate the discovery and design of highly active and stable single-atom motifs for numerous reactions. A combination of advanced characterization techniques and theoretical calculations will deepen our understanding of catalytically active sites and structure–function relationships. However, ex situ characterization often fails to capture the dynamic changes that the catalytic centers undergo during a catalytic cycle, which can overlook atomic-scale phenomena and lead to a number of pitfalls. Therefore, it is vitally important to monitor the catalysts under in situ *or* operando conditions. In this context, Fang et al.^7^ has applied operando X-ray absorption spectroscopy to reveal the dynamic release of near-free-state Pt atoms from a support during the hydrogen evolution reaction. The insights gained in this work could be instrumental in the rational design of high-performing catalysts. Similarly, translating the theoretical results to working catalysts would aid in rationally designing efficient and robust catalysts for targeted reactions. Guided by density functional theory calculations, Ouyang et al.^8^ demonstrate that the surface coverage of CO molecules on PdAu alloys can transition the catalyst between a single-atom alloy and a Pd cluster structure in situ. Accordingly, the in situ control of active site geometries and composition in PdAu single-atom alloy catalyst can redefine the reaction pathways of ethanol dehydrogenation directly.

Finally, great efforts have been devoted to expanding the application of SACs in industrially important reactions. And thus far, SACs have shown compelling performance for a number of lab-scale reactions under industrially relevant conditions. For example, Yang et al.^9^ creatively designed a single-atom nickel-decorated porous carbon membrane catalyst to sustain a high CO partial current density of 308.4 mA cm^−2^ and 88% Faradaic efficiency for up to 120 h. Meanwhile, Sun et al.^10^ engineered Pt single atom on copper support for propane dehydrogenation with high selectivity and stability for more than 120 h of operation at 520 °C, surpassing significantly the benchmarked Pt nanoparticle catalysts under the same reaction conditions.

While this field has made stunning progress over the past two decades, there is certainly more to achieve. Particularly, it will be fascinating to follow how newly generated knowledge and developed systems are going to make an impact on industrial and commercial levels. We look forward to reading, learning, and disseminating advances in this area over the coming years.
